# An Adaptive Motivation Approach to Understanding the ‘How’ and ‘Why’ of Wellbeing

**DOI:** 10.3390/ijerph191912784

**Published:** 2022-10-06

**Authors:** Reuben D. Rusk

**Affiliations:** Centre for Wellbeing Science, University of Melbourne, Melbourne, VIC 3010, Australia; reuben.d.rusk@mindquip.com

**Keywords:** happiness, evolutionary psychology, computational neuroscience, theory, adaptation, motives, emotion, negativity bias, variety

## Abstract

A new model provides insight into the ‘how’ and ‘why’ of wellbeing to better understand the ‘what’. Informed by evolutionary psychology and neuroscience, it proposes that systems for adaptive motivation underpin experiential and reflective wellbeing. The model proposes that the brain learns to predict situations, and errors arise between the predictions and experience. These prediction errors drive emotional experience, learning, motivation, decision-making, and the formation of wellbeing-relevant memories. The model differentiates four layers of wellbeing: objective, experiential, reflective, and narrative, which relate to the model in different ways. Constituents of wellbeing, human motives, and specific emotions integrate into the model. A simple computational implementation of the model reproduced several established wellbeing phenomena, including: the greater frequency of pleasant to unpleasant emotions, the stronger emotional salience of unpleasant emotions, hedonic adaptation to changes in circumstances, heritable influences on wellbeing, and affective forecasting errors. It highlights the importance of individual differences, and implies that high wellbeing will correlate with the experience of infrequent, routine, and predictable avoidance cues and frequent, varied, and novel approach cues. The model suggests that wellbeing arises directly from a system for adaptive motivation. This system functions like a mental dashboard that calls attention to situational changes and motivates the kinds of behaviours that gave humans a relative advantage in their ancestral environment. The model offers a set of fundamental principles and processes that may underlie diverse conceptualisations of wellbeing.

## 1. Introduction

Different individuals, groups, literatures, and cultures conceptualise wellbeing differently. There are many *qualitative* definitions of wellbeing, which define it in broad conceptual terms [1,2,3]. There are also many *quantitative* definitions of wellbeing, which describe it in terms of its constituents [4,5,6]. Imagine defining a modern smartphone to someone in 1970 by simply saying it is a hand-held electronic device (a qualitative definition) that consists of a case, battery, electrical components, and glass (a quantitative definition). Such a definition fails to convey the important knowledge of how it functions and why it is useful, and wellbeing definitions often fall short in a similar way. There are relatively few *functional* definitions that capture the essence of how wellbeing operates and why it operates as it does. Functional definitions have potential to ground the many different conceptualisations of wellbeing within basic processes that transcend their differences.

Psychological approaches have sketched coarse outlines of the ‘how’ and ‘why’ of wellbeing based on psychological theories and research findings [7,8]. Neuroscientific approaches have described the brain structures and neurophysiological processes involved [9,10,11]. Mathematical approaches have attempted to model the temporal dynamics of wellbeing [12,13]. Evolutionary approaches have described functional specialisations in the brain that relate to wellbeing [14,15,16,17]. No single existing approach appears sufficiently connected with psychological research, neuroscience, mathematics, and evolutionary principles to provide a compelling understanding of the ‘how’ and ‘why’ of wellbeing. The shortcomings of each approach are visible in contrast to the strengths of the others.

To address this gap in wellbeing theory, this paper will put forward a model that attempts to capture important features of how and why wellbeing functions, in which each approach has a place and scope to refine details. It will first introduce the model from a theoretical perspective, before integrating existing literature into the model. Following sections will describe a numerical implementation and qualitative results used to investigate whether the model has potential to reproduce important characteristics of wellbeing. The results will be discussed in the context of empirically researched wellbeing phenomena to explore the implications of the model and how it aids in understanding the nature of wellbeing.

### 1.1. Model Overview

**Figure 1:**
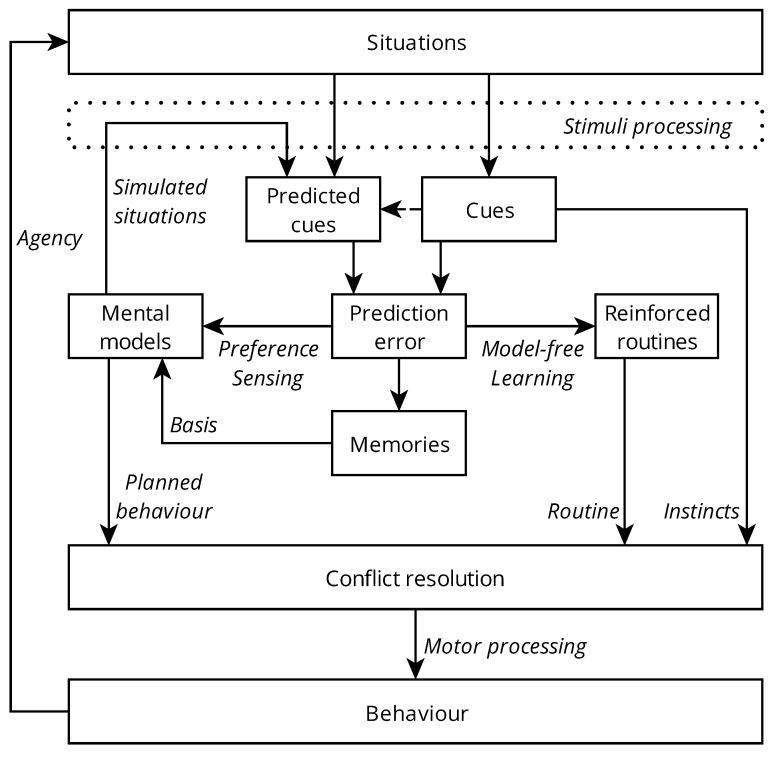
The Adaptive Motivation Model. Arrows represent influence.

Figure 1 shows an overview of the proposed Adaptive Motivation Model. The model is based within an evolutionary perspective. Natural selection occurs in populations when heritable variations in phenotypes (traits, in this case) allow certain individuals to pass on their phenotypes to future generations at a higher rate than others. The degree to which an individual’s phenotypes are reproduced in the next generation will be termed fitness. Individuals with higher relative fitness reproduce more those with lower fitness, and their descendants come to dominate the population. This principle forms the backdrop for the model.

### 1.2. Situations, Behaviour, and Agency

A fundamental attribute of humans, and indeed organisms in general, is that they have agency. They can alter their fitness through their behaviour. One behaviour might find a new source of food to increase fitness, while a different behaviour may result in an injury that decreases it. These situations change fitness in different ways, and behaviour can influence which situation eventuates. Moreover, humans can change their environment to increase opportunities and reduce threats (niche-construction), and this process may play an important role in wellbeing [18,19].

The fundamental challenge of having agency is in using it effectively with limited resources of time and energy. The complexity and difficulty of maximizing fitness through agency within a complex environment is hard to overstate. The challenge inherently concerns motivation, since it guides behaviour [20,21]. Motivation is the focal point of the model.

### 1.3. Cues

Situations offer a very large number of potential stimuli, and adaptive behaviours must be situation-specific. With a limited capacity to process these stimuli, humans can only give cognitive resources to a subset of them. If left to chance, individuals might miss important cues. So, certain genetic and non-genetic predispositions could give individuals an advantage.

The model assumes individuals develop predispositions to attend to and process particular stimuli in certain ways, which result in certain neural patterns of activity. The brain includes a range of functionally specialised structures. For example, lower-order processes operate quickly and effortlessly to distil particular sets of physical stimuli into more compact representations [22]. Other processes detect certain sets of social stimuli [23,24]. For example, these might include detecting losses or gains of social support [25]. More complex, higher-order patterns may develop through low-level learning processes from a small initial set of simple predispositions. This developmental cascade is likely shaped by a diverse range of genetic, environmental, social, cultural, and chance influences.

This paper will term these neural patterns *cues*. Situations elicit certain cues depending on how these neural patterns have developed in the individual under heritable influences. Functionally, cues serve as the detectors of fitness-relevant physical signals, situation characteristics, or types of situations [26]. Stimuli for these cues could also include interoceptive awareness of internal states and processes. Simple examples are hunger or tiredness cues, which have a strong physiological basis. Humans have some awareness of their own thoughts and emotions, which also provide remarkably useful cues [25].

Cues function as meaningful inputs for motivational processes, and the quality of motivation depends on the quality of these inputs. Evolutionary psychology suggests that selection pressure applies to them. Individuals who generate higher quality cues would have a reproductive advantage from an evolutionary perspective. The cues most useful as motivational inputs will relate to situations that either increase or decrease fitness, so these are likely to be what adapted individuals attend to.

Cues can be separated into two types. Outcome cues correspond with increases or decreases in fitness that are not contingent on behaviour. Predictive cues correspond with possible future changes in fitness that are contingent on situation-specific responses (i.e., opportunities and threats). Both cue types provide useful signals for motivation.

### 1.4. Cue Value

The model assumes that cues have a neurological value (*cue value*) that determines how they subsequently influence motivation. Such values distinguish affective phenomena from other non-affective phenomena [27]. *Approach* cues (positive) increase the likelihood of behaviours that make those cues more likely to reoccur, while *avoidance* cues (negative) do the opposite.

Since the domains for cue value are so diverse and outcomes are so complex, no general purpose cognitive system can readily assign appropriate values [23]. Instead, it has been suggested that this value system is generated by functionally specialised and context-specific brain systems shaped through generations by selection pressures [25]. Current physiological states may modulate cue values (for thirst, for example).

Individuals presumably develop values to certain cues through the same complex developmental cascade that gives rise to the cues themselves. Higher-order cues can become associated with the value of the lower-order cues, and thus expand upon an initial set of simple cues to deal with greater and greater stimuli complexity. A similar development of increasingly complex behaviours likely occurs in parallel, allowing increasingly complex behavioural responses.

### 1.5. Prediction Error

The brain continuously and automatically makes predictions about the future based on learned associations [28]. It can thus continuously calculate *prediction errors*, which represent the differences between actual and expected outcomes. This model proposes that the brain calculates prediction errors based on the difference between the predicted value of a cue and the value experienced when it occurs. These predictions are context-specific and depend on previous experiences.

Neuroscience has now amassed considerable evidence that the brain calculates prediction errors [29,30,31,32]. Evidence suggests these play a key role in learning and decision-making [30,33,34]. Prediction errors drive learning about approach cues [31]. A similar error-based process may drive learning to mitigate avoidance cues [35,36], although different brain structures may be involved [33].

A large body of work suggests predictions and prediction errors are ubiquitous throughout the brain [37]. Recently, neuroscientists have given increasing attention to predictive process models of various neurological phenomena [38]. Recent models view the brain as a dynamic, hierarchical, inference system and suggest predictions and prediction errors form the basis of emotional experiences [27,37], which some recent psychological research supports [39]. Along similar lines, the model proposes that prediction errors elicit experiences of emotion. The larger the prediction error, the stronger the emotion.

### 1.6. Motivation

According to Aunger and Curtis [40], the brain has three motivational systems. They termed these ‘reflexive’, ‘motivated’, and ‘planned control’, but this paper will term them *instinct*, *routines*, and *planned control*, respectively. They each provide increasingly powerful mechanisms to adapt behaviour, and the model bases these mechanisms on cues and cue values.

#### 1.6.1. Instincts

Instincts represent a biological preparedness that makes certain processes or behaviours more or less likely in the context of certain cues. They are *if-then* responses to cues, and cue value may play less of a role. Instincts relate to basic physiological concerns, such as food, reproduction, and safety. Each instinctive motivation will increase or decrease fitness by biasing behavioural responses in particular contexts. They likely play their most important role in the earliest stages of life, before much learning has occurred.

Instincts, almost by definition, fulfil the conditions for natural selection and are subject to evolutionary pressures. Individuals with more adaptive instincts will have an evolutionary advantage over those will less adaptive instincts. Hence, the instincts of individuals within an adapted population will tend to increase fitness.

#### 1.6.2. Routines and Reinforcement Learning

In this model, routines are sets of behaviours that become reinforced in specific contexts. Thus, on exposure to a familiar set of predictive cues, an individual can automatically execute a reinforced behavioural routine. Some routines might increase fitness, while others might decrease it. Reinforcing particular routines thus plays a critical role in fitness. According to reinforcement-learning theory [41,42], reinforcement learning is driven by prediction errors. So, the model assumes that prediction errors make context-specific routines more or less likely through reinforcement learning.

Certain inherited cue values lead to adaptive reinforcement learning, and give individuals a reproductive advantage. Other cues will do the opposite. Hence, within an adapted population, cue values will tend to correspond with how the situations that elicit them influence fitness. Situations that increase fitness will tend to elicit approach cues, while situations that decrease fitness will tend to elicit avoidance cues. Members of an adapted population will tend to share similar values for a similar set of cues.

#### 1.6.3. Planned Control

Planned control represents the third and most powerful motivational mechanism. Central to this mechanism is the development of mental models of the world that can accurately predict future possibilities. Neuroscientific research suggests that prospection plays a critical role in planned behaviour [43,44]. Possible futures are mentally simulated, which are then used to determine the context-dependent values of potential options to guide choice-making [45,46].

Based on such research, the model assumes that mentally simulated situations serve as inputs into an already well-developed cue-based motivation system. Simulations can elicit meaningful cues and their associated values, which can generate errors between the value of those options and current expectations. These error values could thus serve as the preference signals (i.e., values) in heuristic choice-making processes to guide adaptive behaviour [22]. Neurological signals of prospection presumably allow for differentiated processing of real and simulated cues.

The model proposes that a hierarchy of goals emerges as mental models of the world develop increasing levels of abstraction and complexity. Base-level goals correspond with the simplest of choices. Such goals relate closely to particular cues, and can be informed directly by preference sensing. Lower-order goals become the building blocks of higher-order goals. Very high-order goals, such as “get a promotion”, rest on a hierarchy of lower-order goals, making them more detached from base-level cues and preferences. The processes and outcomes of these high-order goals are more complex. Thus, achieving some high-order goals may change the cues experienced, but achieving others may not.

### 1.7. Conflict Resolution

Different combinations of cues may elicit impulses toward multiple incompatible behaviours. Thus, individuals must often suppress impulses for competing behaviours. Based on neuroscientific and psychological research [47,48], the model assumes that executive functions such as response-inhibition and self-regulation resolve these motivational conflicts.

This conflict resolution function may be critical in bringing together many of the functionally specialised processes within the brain. Many distinct brain processes could be independently involved in the generation of cues, cue value, prediction errors, and learning processes [23]. The conflict resolution function might allow the behavioural outputs of such different processes to be harmonized.

### 1.8. Memory and Forgetting

Research in neuroscience has shown that prediction errors play an important role in episodic memory formation [49,50,51]. Several models posit that learning occurs when the experienced reward (i.e., value) differs from expectations [50,52,53], although the reward value itself also plays a role [54]. Neuroscientific evidence suggests that the episodic memories formed in this process also serve as the basis for prospective simulation of future events using partially shared neurological structures [44,55,56]. In the model, each prediction error leads to a memory that in some sense has a value matching that prediction error.

Neuroscience also suggests that memories decay, which may ensure the most useful information is retained. The decay involves several processes, including interference from new memories and a process of ‘active forgetting’ [57,58]. Moreover, experimental research has shown that retrieval probabilities for long-term memories tend to decay as a power function of time [59,60,61]. Based on such research, the present model assumes that predicted cue values, routine strengths, and memory values slowly decay as a power function of time. Computational models of memory have often assumed such temporal decays, although debate continues about the mechanisms [62,63]. Temporal decay may help behaviour to adapt to changing circumstances. It also matches well with everyday human experience. Cues that are repeated often will lead to more and more accurate predictions and smaller errors, gradually producing weaker affective responses. In contrast, unexpected cues in infrequent, surprising, or uncertain situations will have larger prediction errors and hence stronger affect.

### 1.9. Wellbeing

The Adaptive Motivation Model serves as a lens through which to understand the nature of wellbeing. It suggests four related layers of wellbeing: objective, experiential, reflective, and narrative. Each layer is deeper and more complex than the previous ones, as summarised in Table 1. Seven aspects of wellbeing have been listed, which will be covered in more detail in subsequent sections. Each aspect relates to each layer in different ways.

First, objective wellbeing depends on the recent situations and behaviours of the individual as objectively assessed, not on any internal processes. One can assign subjective values to situations within the context of a particular value system (physical health, evolutionary fitness, etc.), which the individual may or may not share. When assessed as a whole, these values constitute a measure of objective wellbeing, such as observed quality of life.

Second, experiential wellbeing is the moment-by-moment experience of psychological affect that prediction errors elicit, along with their emotional significance. Cues that prompt positive errors motivate the individual to *want* them. On the other hand, cues that prompt negative errors motivate the individual to *not want* them. This differentiation may form the basis for a subjective significance of the cues, such as positive or negative, good or bad, and so on.

Reflective wellbeing is constructed consciously from readily recallable memory representations, with the most recent memories and those associated with the strongest emotion tending to dominate [64,65]. The reflection process can elicit an emotional sense of the extent to which recent experiences were positive or negative. This process relates to the affective component of subjective wellbeing, which Diener et al. [66] defined as a person’s cognitive and affective evaluation of his or her life. Hedonic wellbeing [3] relates to reflective wellbeing.

Finally, narrative wellbeing relates to the cognitive significance of narratives and beliefs developed from past experiences. It relates to the cognitive aspect of subjective wellbeing and an assessment of one’s own life satisfaction. Individuals frame wellbeing-relevant memories within a more cohesive narrative that emphasises certain memories over others and infuses them with meaning. A hierarchy of narratives may emerge from episodic memories, with higher-level narratives allowing the influence of past events to persist for longer periods [67]. Consequently, narrative wellbeing may have a much longer time-frame and greater selectivity than reflective wellbeing.

The same set of memories can serve as the building blocks for many different narratives that differ in their subjective qualities. Hence, narrative wellbeing is tied to experiences more loosely than the lower wellbeing layers. From the perspective of wellbeing, events from the more distant past have particular relevance to beliefs and evaluations that relate to the self.

Eudaimonic wellbeing [3] relates to both objective and narrative layers in different ways. As an observation, it concerns the objective layer, since it evaluates situations and behaviours within an external value system. As a reflective self-assessment, eudaimonic wellbeing relates to the narrative layer, since it imbues autobiographical memories with cognitive significance within the framing of certain narratives and beliefs.

The suggested aspects of wellbeing have relevance to each layer in different ways. For example, a meaningful event can be observed objectively (objective layer), it often elicits emotions (experiential), it can be ‘re-lived’ in the near term through recall (reflective), and it may influence self-narratives and beliefs (narrative). Many higher-level cues impact on wellbeing at all four levels. However, lower-level cues that are more sensory in nature relate more strongly to the experiential layer, since they may be quickly forgotten or not incorporated into narratives.

### 1.10. Proposed Integration with Existing Literature

A good model integrates with existing literature. This section will explore how the Adaptive Motivation Model integrates with literature through three lenses: existing terms that relate to wellbeing, human motives, and specific cues and emotional experiences.

#### 1.10.1. Wellbeing Terms

The model suggests the prediction errors that underlie wellbeing would have guided humans in the ancestral environment to increase and maintain fitness by shaping their motivations. So, the first lens connects fitness with quantitative definitions of wellbeing.

Based on the reviews of Hone et al. [4], Longo et al. [5], and Martela and Sheldon [6], Table 2 shows terms relevant to wellbeing (i.e., wellbeing constituents) within nine aspects. The nine aspects were developed iteratively by the author to thematically group all the wellbeing terms identified in these reviews. Each have proposed associations with changes in fitness. For clarity, Table 2 describes only associations for increasing fitness (the ‘positive’ direction), and implies their opposites for decreasing fitness.

The model suggests positive and negative emotions are intimately linked with changes in fitness and prompted by many different kinds of cues. The first seven aspects organise these kinds of cues into thematic groups. Cues may relate to more than one aspect, and the aspects listed here may not be comprehensive. The eighth aspect, positive emotion, is general and relates to the experiential and reflective layers of wellbeing. Cues within the first seven aspects often elicit positive emotions, but the positive emotion aspect could also include any cues that do not fit within those first seven aspects. The final aspect, positive narrative, involves self-referential narratives and beliefs. It relates to the narrative layer of wellbeing. The associations between terms in this last aspect and fitness, if present, seem more indirect.

The aspects in Table 2 serve to group together different kinds of cues thematically, and do not imply that each is subserved by a common underlying neurological process. In the model by Martela and Sheldon [6], aspects such as autonomy, competence, and positive relationships (relatedness) are viewed as psychological needs. The approach here does not imply high-level needs drive cues, but rather implies that cues underlie needs. Many cues share common themes from which aspects can be formed. Categorisation provides a convenient way to describe a diverse range of cues, but different categorisations are possible.

Table 2 offers only a starting point to link various facets of wellbeing to changes in fitness, at least within an ancestral human environment. With space in mind, this paper will leave elaboration and validation of such associations for elsewhere.

#### 1.10.2. Motives

If motivational processes underlie wellbeing, strong links might exist between the aspects in Table 2 that relate to wellbeing and common human motives. Along these lines, Table 3 relates these aspects to motives identified by several researchers. Different authors organised these motives into different hierarchies, which differed from the groupings in Table 3. Nevertheless, most motives correspond at face value with one or two aspects. The model suggests certain cues underlie these motives.

For some motives, the connection with wellbeing only becomes clear by shifting the reference frame to receiving the behaviour, such as control of others or dominance. Motives concerned with positive expectancy often involve environmental factors such as safety and security. Environmental factors impact several wellbeing aspects, however.

Significantly, motives relating to mating and resources did not fit well with the seven main wellbeing aspects in Table 2. Each relates to fitness, at least in the ancestral environment. The adaptive motivation perspective implies that they may also influence wellbeing in some circumstances. Empirical data suggests the mating domain does. For example, widowhood influences wellbeing significantly, and a large percentage of people report lasting changes in subjective wellbeing after marriage [68,69]. The effect of money is more complex, perhaps because money itself is poor at eliciting cues that adapted humans detect and respond to. That said, greater wealth correlates with greater wellbeing, particularly above a minimal level, since it aids need fulfilment [70]. As wealth increases, its marginal effect on wellbeing diminishes [71].

**Table 2:** Proposed associations between wellbeing constituents and fitness by wellbeing aspect.

Wellbeing Aspect	Proposed Association with Fitness	Wellbeing Terms
Vitality	Physically capable of agency to increase fitness	Vitality [5,72,73]
		Energy [74]
Engagement	Behaviour is likely to be increasing fitness (through learning, skill development, use of skills, etc.)	Interest [75]
		Engagement [72,73,76,77]
		Involvement [5,78]
		Effort in pursuing excellence [78]
		Enjoyment [78]
Positive relationships	Increase in fitness due to social factors	Positive relationships [72,75,76,77,79]
		Relatedness [80]
		Connection [5]
		Social belonging/trust [5,73]
		Supportive relationships [73]
Autonomy	Fitness is less limited by dominance of others	Autonomy [73,74,75,79,80]
		Self-congruence [5]
Competence	A particular type of agency will be more effective in increasing fitness	Competence [73,74,76,80]
		Accomplishment [77]
		Environmental mastery [79]
		Self-esteem [72,73,76]
		Manageability [81]
		Comprehensibility [81]
		Clear thinking [74]
Positive expectancy	Fitness is more likely to increase in the future, or not decrease	Optimism [5,72,73,76]
Meaning	Planned behaviour is/was worthwhile to increase fitness, (particularly through social support)	Purpose [5,73,75,76,77,78,79]
		Meaning [5,72,73,77,78]
		Meaningfulness [81]
		Significance [5]
		Contribution [75,76]
*Positive emotion* (Experiential and reflective layers, covering all cues)	General increase in fitness, or absence of decrease	Positive emotion/feelings [73,76,77]
		Happiness [5,75]
		Emotional stability [72]
		Calmness [5]
		Absence of negative feelings [73]
*Positive narrative* (Narrative layer, covering all cues)	Indirect	Self-acceptance [5,74,75,76,79]
		Self-worth [5]
		(Personal) Growth [75,79]
		(Personal) Development [5,74,78]
		Self-discovery [78]
		Satisfying life [73]
		Resilience [5,73]

**Table 3:** Proposed links between wellbeing aspects and motives.

Aspect	Motives
Vitality	Health and fitness, physical strength and endurance [82]; acquiring metabolic resources (1), maintaining body, avoiding infection (1) [40]; physiological needs [83]; health [84]; healthy, clean [85]
Engagement	Curiosity and exploration (1), mental knowledge and skills (1) [82]; acquiring knowledge about the world (1), honing skills (1) [40]; exploration, appreciating beauty, effort [84]; creativity, curiosity [85]
Positive relationships	Affection/commitment, altruism, social exchange, curiosity and exploration (2) [82]; affiliation, status improvement, maintaining functioning of large non-kin groups [40]; affiliation, status/esteem, parenting [83]; social values, social giving, interpersonal care, respect, avoiding rejection, interpersonal effectiveness, socialising, social life and friendship, being liked, being close to parents’ family [84]; social recognition (1), sense of belonging, politeness, honouring of parents and elders, devoutness, humility, helpfulness, forgiving, honest, loyal, mature love, true friendship, equality, social justice [85]
Autonomy	Dominance and aggression (giving or receiving) [82]; control of others, leadership, confidence and autonomy, avoiding conflict [84]; freedom, independence, choosing own goals, authority, social power, obedience, respect for tradition, accepting one’s portion in life [85]
Competence	Mental knowledge & skills (2) [82]; honing skills (2), acquiring knowledge about the world (2) [40]; self-knowledge, fastidiousness, mastery and perseverance, avoiding failure, self-regulation, smartness and rationality, organisation and efficiency, analysis & technical know-how, intellectual growth, occupational success [84]; self-respect, ambition, influence, capability, success, intelligence, self-discipline, wisdom, broad-mindedness [85]
Positive expectancy	Safety [82]; avoiding predation, avoiding infection (2) [40]; self-protection [83]; avoiding harm, stability and safety [84]; national security, family security, social order, moderation, protecting the environment, a world at peace [85]
Meaning	Legacy, meaning [82]; inspiring others, wisdom and serenity, pursuing ideas and passions [84]; social recognition (2), responsibility, meaning in life, inner harmony, detachment, unity with nature, a world of beauty [85]
*Mating*	Sex, appearance [82]; mating, acquiring high quality sexual relationships, maintaining high quality sexual relationships [40]; mate acquisition, mate retention [83]; Sexual intimacy [84]
*Resources*	Wealth [82]; acquiring metabolic resources (2), accumulating surplus resources [40]; money and wealth, financial freedom [84]; wealth [85]
*Positive emotion*	Happiness, avoiding stress and anxiety [84]; pleasure, daring [85]
*Positive narrative*	Enjoying life, religion and spirituality, being better than others, personal morals [84]; enjoying life, an exciting life, a varied life, a spiritual life, public image [85]

#### 1.10.3. Cues and Emotions

Table 4 gives several examples of how relatively specific cues and emotions might contribute to the more general wellbeing aspects. It illustrates how the Adaptive Motivation Model can relate to a wealth of existing research that already links specific experiences and emotions to wellbeing. Since relative fitness matters, adapted individuals might also be particularly sensitive to cues relating to relative advantage or disadvantage. For example, a lack of kindness may have more significance if others receive kind treatment.

## 2. Methods

This section outlines a simple computational implementation of the model (see the Supplementary Material). The purpose of this model was to qualitatively investigate the predicted wellbeing dynamics of the model so they could be compared against existing empirical research. The Python model code and documentation are publicly available in the *pywellbeing* package at https://pypi.org/ and https://github.com/ (accessed 29 August 2021). The implementation required no external data sources. Since all variables were represented abstractly as numerical values, they did not require operationalisation.

### 2.1. Plain-Language Overview

The numerical implementation simulated how individuals behaved, learned, and reproduced. It rested on a set of independent situations, which differed only in their frequency and how they influenced fitness. Situations that could slightly influence fitness occurred often, while those that could greatly influence it occurred rarely. The choice between two behaviours could avert the outcome of a situation. Individuals learned which choices to make through a numerical representation of the Adaptive Motivation Model. The implementation needed no particulars of individuals, situations, or cues, as they were generic. Starting with a ‘naive’ generation, the simulation allowed individuals in each generation to learn and make motivated choices over many periods, which led to certain levels of fitness and wellbeing. Each subsequent generation was bred from only the individuals with higher relative fitness, which simulated natural selection pressure. Adaptive motivational predispositions could thus develop over successive generations.

### 2.2. Situation Frequency

The numerical simulation was based around a set of n=80 situations that elicited predictive and outcome cues, which each could potentially change fitness by an amount, $z_{i}$. Situations were generic, and differed only in their $z_{i}$ values. Each simulated individual was exposed to m=200 time periods with these 80 situations. The base frequency of the individual encountering a given situation, i, in the environment was assumed to follow a standard normal probability distribution, given by:

(1) $$p_{i}=\varphi \left(z_{i}\right)=\frac{e^{-z_{i}^{2}/2}}{\sqrt{2\pi }}$$

where $p_{i}$ is the frequency of a given situation that can change fitness by $-2\leq z_{i}\leq 2$. Thus, frequent situations could cause small changes in fitness, while less frequent situations could cause larger changes. To simulate the effect of niche-construction for each individual, planned behaviour was assumed to modify the frequency of encountering each situation for each period, k, so it became:

(2) $$q_{ki}=A^{\phi_{ki}}\left(p_{i}\right)$$

Here, $\phi_{ki}$ was an ‘effort’ value that corresponded to the degree to which individual successfully increased or decreased the frequency of the situation. The constant A was chosen to be 5, and $q_{ki}$ was the resulting frequency of encountering the situation. Positive effort values increased the frequency of the situation, and negative effort values decreased it.

### 2.3. Motivational Processes

Instincts and reinforcement learning determined the likelihood of two behaviours in response to predictive cues elicited by each situation. One behaviour nullified the situation so there was no outcome cue and no impact on fitness, while the alternative behaviour allowed the outcome to occur and fitness was changed by $z_{i}$. The probability of the latter behaviour, $b_{ki}$, was given by a logistic function of the instinct and learned reinforcement values for the situation in the current period, $G_{ki}$ and $R_{ki}$ (respectively), as follows:

(3) $$\;b_{ki}=f\left(G_{ki}\;+\;R_{ki}\;\right)\;=\;\frac{1}{1\;+\;e^{-\left(G_{ki}\;+\;R_{ki}\;\right)}}\;$$

The probability of the former behaviour was thus $1-b_{ki}$. This approach avoided the need for complex conflict resolution logic while accounting for both instincts and reinforcement learning.

Instinct value, $G_{ki}$, and cue value, $V_{ki}$, for each cue were inherited at random from the individual’s parents with some modification. Namely, the inherited values were capped to a magnitude of 1.5 to prevent instincts and reinforcement becoming overly effective at mitigating the probability of negative outcomes. A randomly generated value with a standard deviation of 0.4 was then added to these capped values. The first generation began with only this random component.

For each situation at each period, prediction error, $\varepsilon_{ki}$, was calculated as:

(4) $$\varepsilon_{ki}\;=\;V_{ki}\;-\;\lambda_{ki}\;$$

where $\lambda_{ki}$ was the predicted value, which always began at 0 for the first period. This error was weighted to reflect how frequently the individual would experience the outcome of the situation, resulting in a weighted prediction error, $\omega_{ki}$.

(5) $$\omega_{ki}=\;b_{ki}q_{ki}\varepsilon_{ki}\;$$

Based on standard approaches to update predicted values [33], the weighted prediction error was used to update the predicted value for the next time period, $\lambda_{k+1,i}$, given by:

(6) $$\lambda_{k+1,i}=\gamma_{e}\left(\lambda_{ki}\;+\;\alpha_{e}\omega_{ki}\right)\;$$

where $\alpha_{e}=2$ was a learning rate and $\gamma_{e}=0.99$. The learned reinforcement value for the next period, $R_{k+1,i}$, was:

(7) $$R_{k+1,i}=\gamma_{g}\left(R_{ki}\;+\;\alpha_{r}\omega_{ki}\right)$$

where $\alpha_{r}=3$ was a learning rate and $\gamma_{g}=0.98$.

Calculation of effort values that governed planned control was as follows. Effort for each situation, $\phi_{ki}$, began at 0 for the first period (k=0). If the sum of absolute effort values across all situations did not exceed a limit of 8.0, effort was calculated as:

(8) $$\phi_{k+1,i}\;=\;\phi_{ki}\;+\;\alpha_{c}\omega_{ki}\;$$

where $\alpha_{c}=0.01$. If the summed absolute effort values exceeded κ=8.0, it was assumed that the individual could not exert more agency and thus could only reallocate effort between situations.

Effort reallocation by each individual at each time period, k, was done by calculating 20 ‘decisions’. In each decision, u, two situations, i and j, were chosen at random. The marginal effect on weighted prediction error of swapping effort between these two situations, $\Delta_{ku}$, was calculated as follows:

(9) $$\Delta_{ku}=\left(A^{\phi_{ki}+\alpha_{c}s\left(\phi_{ki}\right)}\;-\;A^{\phi_{ki}}\right)\omega_{ki}+\left(A^{\phi_{kj}-\alpha_{c}s\left(\phi_{ki}\right)}\;-\;\;A^{\phi_{kj}}\right)\omega_{kj}\;$$

where $s\left(\phi_{ki}\right)$ was the sign of $\phi_{ki}$. The left term estimates the marginal change in prediction error from increasing effort for situation i and the right term estimates the marginal change in prediction error from decreasing effort for situation j. If $\Delta_{ku}>0$, effort for situation i was changed by $\alpha_{c}s\left(\phi_{ki}\right)$ and effort for situation j was changed by $-\alpha_{c}s\left(\phi_{ki}\right)$, and visa versa if $\Delta_{ku}<0$.

### 2.4. Fitness and Reproduction

A population of S=300 individuals was used for each generation. Once a given generation had reached the end of 200 time periods, the fitness for each individual, $F_{y}$, was calculated as:

(10) $$F_{y}\;=\;\overset{m}{\underset{k=0}{\sum }}\overset{n}{\underset{i=0}{\sum }}b_{ki}q_{ki}z_{i}\;$$

which reflects the assumption made earlier about how the situations can increase or decrease fitness. To reduce computation time, strong selection pressure was imposed. In each generation, the highest 10% of individuals in terms of fitness were randomly selected to mate with the highest 60%, again selected at random, to create the next generation of 300 individuals. A total of 80 generations were simulated.

### 2.5. Wellbeing

Reflective wellbeing for individual y, $W_{yk}$, was calculated considering the prediction errors for a given period.

(11) $$\;W_{yk}\;=\;\frac{\sum_{i=0}^{n}\omega_{ki}f\left(\omega_{ki}\right)}{\sum_{i=0}^{n}\left|\omega_{ki}\right|\;}\;$$

where $f\left(\omega_{ki}\right)$ was 1 if $\omega_{ki}\;>\;0$ and 0 otherwise. This represents all positively valued prediction errors for period k divided by the magnitude of all prediction errors for the same period. This measure could vary from 0 to 1 (or 0% to 100%). It provided a reasonable yet simple mathematical approach to quantify the degree to which recent events were positive or negative, in this case during the most recent period.

### 2.6. Determination of Parameters

The nature of this model precluded setting model parameters using empirical data. Thus, model parameters were set manually to ensure the model performed as intended. If $b_{ki}$ became too small for avoidance cues, for example, these cues produced an inadequately strong motivational signal. There were similar consequences if κ or A were too large, but if they were too small, their effects were not easily apparent. If $\alpha_{c}$ was too large, numerical instabilities could occur, but if it was too small, learning did not fully develop in time. The same is true for $\alpha_{e}$ and $\alpha_{r}$. Decay parameters $\gamma_{e}$ and $\gamma_{g}$ needed to be high enough to ensure an appropriate balance against $\alpha_{e}$ and $\alpha_{r}$ to develop reasonable predictions and learned reinforcement values. No effort was made to refine the parameters beyond relatively course values (e.g., 2, 3, 5, 0.01, 1–0.01, 1–0.02) that allowed the model to operate as intended, as modest changes led to qualitatively similar results.

## 3. Results

Selected results of the numerical simulation are described below. More detailed results can be reproduced using the *pywellbeing* package. For the purpose of investigating whether the model may offer insight into wellbeing, the qualitative patterns are more important than the particular values.

### 3.1. Wellbeing and Fitness

**Figure 2:**
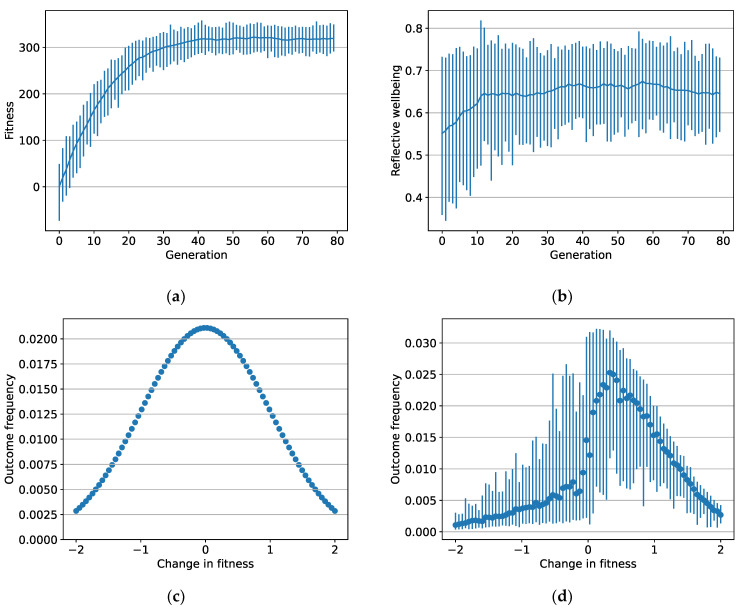
(**a**) Mean fitness across the population for each generation; (**b**) mean reflective wellbeing across the population for each generation; (**c**) frequency of outcomes in the first period of the first generation; (**d**) frequency of outcomes in the last period of the last generation. Points represent population means for the 80 situations, and vertical bars indicate the ranges within the population.

Selection pressure caused the fitness of individuals in each generation to increase significantly and remain steadily high after the 50th generation (see Figure 2a). The increase in fitness corresponded with adaptive motivations, which changed how frequently individuals experienced situational outcomes from Figure 2c during the first period to Figure 2d at the end of the last period. Instinct, reinforced routines, and planned control all played a role, as elaborated further below. Among the adapted population (the 80th generation), the mean wellbeing score was 0.65 at the end of 200 periods, suggesting that individuals were ‘moderately happy’ (Figure 2b).

### 3.2. Instincts and Cue Values

**Figure 3:**
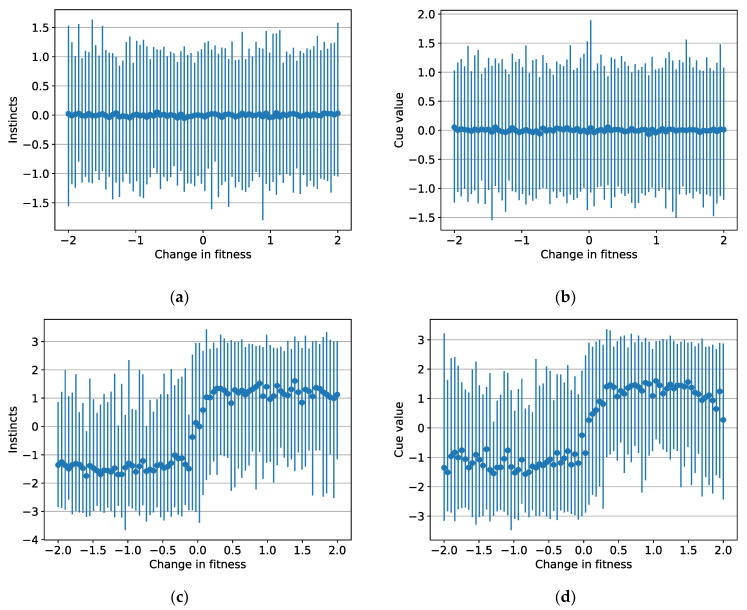
(**a**) Mean instincts for the first generation; (**b**) mean cue values for the first generation; (**c**) mean instincts for the last generation; (**d**) mean cue values for the last generation. Each point represents the population mean for one of the 80 situations, and each corresponding vertical bar shows the range within the population.

The increases in population fitness (Figure 2a) were a consequence of selection-induced changes in heritable instincts and cue values. Figure 3 shows how the population means of these heritable values changed. The constraints on instincts and cue values caused ceiling and floor effects in the means, but considerable ranges in values existed.

Population means for instincts changed from the near-zero values of the initial generation shown in Figure 3a to those shown in Figure 3b for the last generation. Individuals with adaptive instincts gained advantage relative to those with maladaptive instincts and thus came to dominate the population.

Figure 3b shows that, on average, the first generation had no sense of which cues were beneficial or harmful. As a result of selection pressure, the cue values of the last generation (Figure 3d) motivated more adaptive behaviour on average. Reinforcement learning increased the effects on fitness of situations that elicited approach cues and decreased the effects of situations that elicited avoidance cues. Individuals for which cue values corresponded positively with how the situations changed fitness thus benefited from reinforcement learning and gained a reproductive advantage.

### 3.3. Prediction Errors

**Figure 4:**
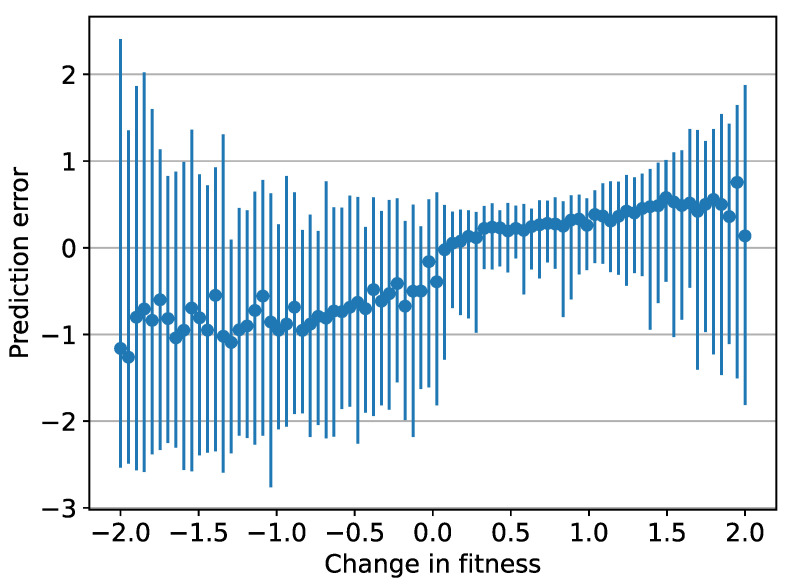
Mean prediction errors at the end of simulation. Each point represents the population mean for one of the 80 situations, and each corresponding vertical bar shows the range within the population.

If prediction errors elicit emotions of corresponding strength, negative cues were significantly stronger than positive cues by 2–3 times for relatively common cues, as shown in Figure 4. Intriguingly, the range of intensity was also much larger for negative cues than for positive cues. Cue values in Figure 3d were mostly similar in magnitude, yet Figure 4 shows that larger changes in fitness corresponded with larger prediction errors and correspondingly stronger emotions. The error prediction mechanism produced stronger prediction errors and emotions for more important situations and cues primarily because they occurred less often. These trends in Figure 4 are notable because individuals had no direct information about how each situation would change their fitness.

### 3.4. Hedonic Adaptation

The numerical model was also used to test the effects of environmental changes. After 200 normal periods, the adapted population was subject to a novel environment in which the likelihood of experiencing a particular cue was much higher. Learning processes for reinforcement learning and planned control processes were disabled at the start of the change to prevent behavioural shifts from influencing the result.

**Figure 5:**
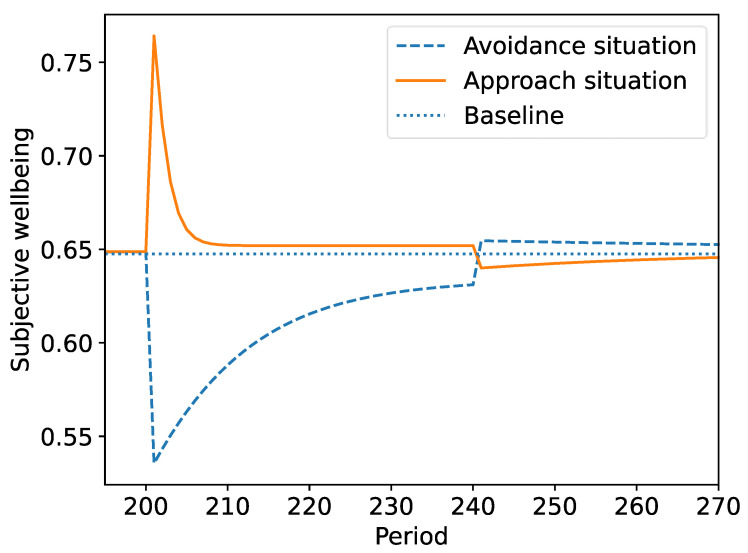
Hedonic adaptation of one individual to a 20-fold increase in the frequency of an approach cue (highest line) or an avoidance cue (lower line) for a duration of 40 periods.

Figure 5 shows example wellbeing histories for an increase in the environmental frequency ($p_{i}$) of either an avoidance situation (at $z_{i}=-1.481$) or an approach situation (at $z_{i}=+1.481$) by a factor of 20 times for a duration of 40 periods. The patterns matched the standard five stages of hedonic response described by Solomon and Corbit [86]. These dynamics arose as predicted values became closer and closer to experiences. Differences in the value for each cue caused differences in the magnitude of responses, which varied between individuals. 

Importantly, while adaptation restored wellbeing to almost the original level, a residual difference remained. This residual difference was a consequence of the decay processes included in the model. Interestingly, individuals tended to adapt to approach cues more quickly and more fully than avoidance cues.

Once the change was reversed after 40 periods, wellbeing levels did not immediately return to baseline levels. After an avoidance cue became much more frequent, the return to normal resulted in a period of slightly higher levels of wellbeing compared to baseline. Approach cues showed similar but opposite effects.

## 4. Discussion

This paper presents a model of the ‘how’ and ‘why’ of wellbeing, based on considerations from evolutionary psychology and neuroscience, to understand what wellbeing is more deeply. According to this model, experiential wellbeing arises as a consequence of motivational processes to change behaviour adaptively by detecting errors between predicted cues and experienced cues. Memories of situations that elicit significant prediction errors then provide a basis for reflective and narrative wellbeing.

### 4.1. Wellbeing Dynamics

Several qualitative results of the numerical implementation correspond with existing research findings. Collectively, they suggest the model may indeed provide a useful understanding of what wellbeing is and why it operates how it does.

First, the model suggests that modern humans, as an adapted population, will experience approach cues more frequently than avoidance cues through agency, as Figure 2d illustrates. Their motivational predispositions will tend to increase their fitness through instincts, routines, and planned control. That agency will mean they experience a greater number of situations that increase their fitness compared with situations than decrease it. The values of the cues these situations elicit will correlate with changes in fitness, with approach cues corresponding with increases in fitness and avoidance cues corresponding with decreases (see Figure 3d). Thus, individuals will tend to experience approach cues relatively frequently compared with avoidance cues. This prediction has some empirical support. For example, Biswas-Diener et al. [87] found positive emotions are reported more frequently than negative emotions, and Trampe et al. [88] found that positive emotions occur about 2.5 times more frequently than negative emotions. The model supports the idea that ‘doing-well’ underlies ‘being-well’, as Martela and Sheldon [6] have suggested.

Second, the model predicts individuals will feel stronger affect for avoidance cues than for approach cues (see Figure 4), which considerable empirical research indicates is indeed the case [89]. Different frequencies of approach versus avoidance cues together with decays in prediction strength explain this effect. Predictions of less frequent avoidance cues decay to a greater extent than the more frequent approach cues, resulting in larger prediction errors for avoidance cues and consequently a stronger affective experience. If it is indeed adaptive for avoidance cues to be felt more strongly, as Baumeister et al. [89] argue, this model provides a plausible mechanism for how this negativity bias may develop even for complex social cues.

Third, the model predicts several hedonic adaptation phenomena, as Figure 5 illustrates. It suggests that individuals adapt to cues because they learn to predict them more accurately, and thus experience smaller prediction errors. The model also predicts that wellbeing will not fully return to baseline after lasting changes in the frequencies of the approach and avoidance cues they experience, which is consistent with empirical findings [68,69,90]. Individuals in the model also adapted faster and more fully to positive changes than to negative changes, which again has some empirical support [91]. While the model shares some similarities to classical theories of hedonic adaptation [92], the current model has a neurological basis and may provide greater explanatory power.

Fourth, the model suggests that to individuals with the highest wellbeing, avoidance cues will seem infrequent, routine, and predictable yet approach cues will seem frequent, varied, and novel. Experiences where approach cues are frequent yet unpredictable could potentially arise in positive social relationships, for example, where others initiate positive interactions. Flow experiences could also provide a source of varied approach cues, since the nature of the experience often has novel elements. Experimental evidence supports the view that novel or varied positive experiences correspond with higher wellbeing [93,94]. A larger variety of approach cues also allows for more time to pass between repeated cues, which gives more time for their predicted values to decay and leads to larger prediction errors. These ideas may offer a deeper understanding of empirical findings by Sheldon and Lyubomirsky [95]. They found that a variety of positive experiences corresponds with higher happiness and also argued that variety and surprise are important for wellbeing.

Fifth, the model implies that wellbeing itself will have heritable variance, since prediction errors are based on heritable cue values. Indeed, several studies have shown wellbeing to have a heritable component [96,97]. Moreover, if cue values are independent, as assumed in the numerical model, different genetic factors could influence particular components of wellbeing, which Archontaki et al. [98] found empirical evidence for. Genetic variability in cue values could also play a role in the differences found in subjective wellbeing and emotional experiences within different ethnic groups [99].

Sixth, the model suggests that individuals will make systematic errors in choices intended to maximise their happiness. In the model, individuals sense preferences based on how the cues elicited by simulated outcomes compare with their *current* situation, not based on the prediction errors those cues will elicit once the individuals are *in* those future situations. If a choice changes the frequencies at which cues occur, the brain will learn to predict them (hedonic adaptation). Thus, their affective impact in the future situations will differ from their predicted affective impact in the present. Several researchers have found these affective forecasting errors occur and have proposed similar mechanisms [100,101]. Perhaps these errors are a consequence of having a motivational system better-suited to maximising fitness than happiness.

Finally, the model has implications for understanding the relationship between goal achievement and wellbeing. It suggests that goal achievement itself does not have a commanding influence on wellbeing. Rather, wellbeing depends on how cues and their predictions change both during goal pursuit and subsequently. Achieving a major goal after a stressful and highly uncertain process may feel temporarily euphoric, and the events lend themselves to a dramatic, triumphant, and memorable narrative. Yet, achieving the very same goal after a calm and predictable process may feel anticlimactic. Frequent and varied approach cues (e.g., competence) during the project could end with it and even add a subjectively negative aspect to accomplishing a valued milestone.

### 4.2. Individual Differences

The model suggests the same cues may influence wellbeing differently between individuals. Cue values will differ between individuals, as the ranges in Figure 3d indicate, so the same cues will elicit different magnitudes of prediction error. These differences may yield different affective experiences and motivational preferences. The numerical simulation showed these effects, with individuals showing differences in both subjective experience and effort allocation. This result implies the ideal mixture of cues will vary between people and correlate with personality. Consistent with this idea, reliable correlations exist between personality and measures of wellbeing [102]. Cue values or the allocation of attention could also change for particular individuals, for example during different life stages, which could change how cues influence their wellbeing.

Different past experiences may result in differences in predicted cue values, which could cause variability in prediction errors. Increasing the frequency of a particular cue by once daily may significantly change wellbeing for an individual who seldom experiences that cue. Yet, the same increase in frequency is unlikely to change wellbeing much for an individual who already experiences it many times a day. The ways in which particular cues influence wellbeing will thus vary across different social, economic, and demographic contexts.

### 4.3. Understanding Wellbeing

The model suggests that wellbeing arises directly from a system for adaptive motivation. This system functions like a mental dashboard that calls attention to changes in the cues that motivate us—the neural signals that guide our choices and behaviour. A complex developmental cascade makes us *want* to increase or decrease the frequency of the very cues that underlie our wellbeing. Like indicators on a vehicle dashboard, the value of these indicators lies in their reliable correlation with outcomes we implicitly care about. The model suggests those outcomes involve *changes in fitness* in our ancestral environment, and wellbeing indicators function to motivate us toward the kinds of behaviours that gave our ancestors a relative advantage.

According to the adaptive motivation perspective, the brain learns to predict the cues elicited by situations. Differences between experience and prediction generate emotion, drive learning, and guide motivated behaviour. Recent memory representations of these differences form the basis for reflective wellbeing. Narrative wellbeing relates to selected memories over a longer time period that often concern the self, so changes in narrative may significantly change self-evaluation.

The cues that underlie reflective wellbeing are based in the environment, culture, and challenges of our ancestors. Many cues from that environment remain relevant today. However, significant discrepancies exist between modern and ancestral environments that present challenges for wellbeing [17,103]. The Adaptive Motivation Model suggests that these discrepancies will cause a different mix of cues and emotions that contribute to wellbeing. A more detailed understanding of these cues may help practitioners support wellbeing more effectively in a modern technological environment.

According to the model, selection pressures have led to modern humans sharing similar predispositions for cues and cue values. This commonality may in part explain why wellbeing is a meaningful construct to measure across all four levels. Researchers find meaningful correlations in how shared cues influence emotion and wellbeing. However, some cues may be important for only smaller subsets of the population, and these cues could potentially relate to concepts not well covered by common measures.

### 4.4. Limitations and Future Research

Given the complexity of wellbeing, any parsimonious model will by necessity omit nuances and fail to capture details. The current model cannot capture many details of how perception, motivation, learning, memory, and wellbeing function. Thus, it does not replace detailed research.

As a model in its infancy, the numerical implementation serves only as an illustration of wellbeing dynamics. Research is needed to refine and validate the processes and parameters of the model. The model could also benefit from more sophisticated neuroscience-based models for attention, learning, and decision-making. For example, approach and avoidance cues could be dealt with in different ways to better represent the different neural subsystems involved.

Future research could identify cues that act as input signals to wellbeing. This could improve understanding of their relationships with different layers of wellbeing. Further work could also improve understanding of how particular cues relate to wellbeing narratives and their psychological significance.

## 5. Conclusions

This paper introduced the Adaptive Motivation Model of wellbeing that draws on evolutionary psychology and neuroscience. A simple numerical implementation of this model produced several wellbeing phenomena that find support within the existing wellbeing literature. These include a greater frequency of positive affect to negative affect, the more powerful emotional salience of negative emotions, hedonic adaptation, effects of variety and novelty on wellbeing, affective forecasting errors, and a genetic influence on wellbeing. The model also suggests how individual differences may play an important role in wellbeing, since the same cues will influence individuals differently. It represents a step toward a parsimonious explanation for the ‘how’ and ‘why’ of wellbeing, as a way to better understand the ‘what’. It suggests that wellbeing arises directly from specific cues used by the motivational system to guide adaptive behaviour. The Adaptive Motivation Model invites new research into specific low-level cues that generate motivational preferences and wellbeing-relevant memories, and further refinements of the model may be worthwhile.

## Figures and Tables

**Table 1:**
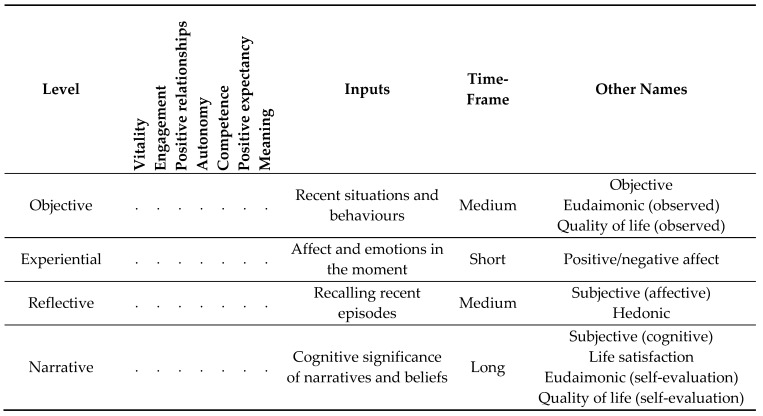
Four levels of wellbeing against various wellbeing aspects. Each aspect applies to each level in different ways.

**Table 4:** Examples of possible cues and emotions for wellbeing aspects.

Aspect	Examples of Possible Approach Emotions or Cues	Examples of Possible Avoidance Emotions or Cues
Vitality	Vigour, strength, speed	Tiredness, physical difficulty, pain, discomfort
Engagement	Interest, curiosity, flow, novelty	Boredom, frustration, overwhelm
Positive relationships	Love, playfulness, touch, gratitude, trust, kindness	Loneliness, grief, deceit, anger, exploitation, exclusion, rejection
Autonomy	Respect, freedom, independence	Coercion, dominance, aggression, obligation
Competence	Goal completion, problem solving, influence	Goal failure, confusion, uncertainty, loss, making mistakes
Positive expectancy	Hopefulness, excitement	Anxiety, fear
Meaning	Praise, appreciation, receiving attention, gratification	Ridicule, embarrassment, criticism

## Data Availability

The data presented in this study was generated using the publicly available *pywellbeing* package as described in the Supplementary Materials section. Data can be reproduced using the *examples.run_amm* function within the package.

## Supplementary Materials

The Python code used for the numerical simulation, together with additional documentation, is publicly available in the *pywellbeing* package at https://pypi.org/ and https://github.com/pythoro/pywellbeing (accessed 29 August 2022).
